# 

**Published:** 1894-07

**Authors:** 


### NOTICES.

The American Dental Society of Europe will hold its nineteenth meeting at Geneva, Switzerland, August 6th, 7th and 8th, 1894. Last year this annual gathering was omitted, as many members wished to attend the Columbian Dental Congress. American colleagues who may be taking a summer vacation-abroad are cordially urged to combine with their plans for travel and sight-seeing a visit to their professional brethren in Europe and to join in the various exercises of the occasion. Members of the profession everywhere will be welcome. Programmes may be had on application to the President, Dr. L. C. Bryan, of Basel, or to the undersigned,

CHAS. W. JENKINS, Secretary
No. 1, Sonnenquai, Zurich.

### EDITORIAL.

### The American Dental Association.

The thirty-fourth annual session of the American Dental Association will be held at Old Point Comfort, Va., commencing at ten o'clock A. M., Tuesday, August 7, 1894.

GEO. H. CUSHING, Recording Secretary.

J. N. Crouse, Chairman of the Executive Committee, 2231 Prairie Ave., Chicago, gives notice that arrangements have been made for a reduction in raiload fare, upon which he says: "We expect a reduction of one and one-third fare for the round trip on the certificate plan. In order to get this reduction full fare must be paid in going to the meeting, and a receipt taken therefor from the ticket agent at the starting point. This receipt must be countersigned by the secretary of the association, at the meeting, and will enable the holder to return for one-third fare, provided one hundred certificates are presented."

To the above we will add that the round trip from Cincinnati to Old Point Comfort will be one fare, fifteen dollars and seventy cents. It may be well from many points west of Cincinnati, southwest and northwest to make arrangements with the roads to Cincinnati, then take advantage of this rate from Cincinnati.

Arrangements will be made to give stop-over tickets at places of resort, as the members may desire.

### National Association of Dental Faculties.

This body will meet at the Hygeia Hotel, Old Point Comfort, on the 4th of August, at ten o'clock A. M., and hold its sessions that day and the 6th.

The Executive Committee of this body will meet at the Hygeia Hotel on Friday, the third of August, at ten o'clock A. M., to prepare business for the general sessions. All interested please make special note of this fact.

### Removal.

The Editorial office of the Dental Register was removed July 1st, 1894, from No. 122 W. 7th Street to No. 12 Berkshire Building corner of Elm St., and Shillito Ave. Also the offices of Drs. J. Taft and Wm. Taft to the same place.

### The Southern Dental Association.

The annual meeting of the Southern Dental Association will be held at Old Point Comfort, Virginia, August 2, 3, 4 and 6, inclusive. A large and interesting meeting is anticipated and a cordial welcome extended to all members of the profession.

Stomatology or Dentistry; which?

### CONTENTS.

COMMUNICATIONS.

### THE DENTAL REGISTER,

A MONTHLY MAGAZINE DEVOTED TO THE INTERESTS OF THE

**DENTAL PROFESSION.**

*Terms Reduced to* $2.00 *Per Year. Single Copies,* 25 *Cents.*

**EDITED BY PROF. J. TAFT, D.D.S.**

—AND—

**Published by SAM'L A. CROCKER & CO., Ohio Dental and Surgical Depot.**

The volume commences in January and closes in December.

The REGISTER being issued monthly is always fresh, therefore Indispensable to the practitioner who desires to keep posted up with the new inventions and improvements which are daily developed. Neither expense nor effort will be spared to give value and variety to its contents. Articles will be illustrated with well executed engravings whenever they will add to the interest or assist to explanation.

Its extensive circulation and frequency of issue make it one of the BEST DENTAL ADVERTISING MEDIUMS in the United States.

All subscriptions must begin with January or July.

Local or special notices, five lines or less, will be inserted for $3 each insertion; over five lines, 50 cts. per line.

All advertising bills payable in advance, or at furthest, after the issue of the first number containing the advertisement. All transient advertisements to be paid for in advance.

☞CONTRIBUTIONS SOLICITED.

Exclusively Editorial Communications should be addressed to the Editor.

The latest number of the REGISTER may be ordered with or without other goods any time, and all letters should be addressed to

